# Nutrition Knowledge and Perspectives of Physical Activity for Pre-Schoolers amongst Early Childhood Education and Care Teachers

**DOI:** 10.3390/nu12071984

**Published:** 2020-07-03

**Authors:** Jeanette Rapson, Cathryn Conlon, Ajmol Ali

**Affiliations:** School of Sport, Exercise and Nutrition, Massey University, Auckland 0632, New Zealand; J.Rapson@massey.ac.nz (J.R.); C.Conlon@massey.ac.nz (C.C.)

**Keywords:** childcare, day care, nutrition knowledge, pre-school teachers, obesity prevention

## Abstract

Caregivers’ nutrition and physical activity knowledge is recognised as being important for children’s health and body size. Identifying knowledge gaps amongst caregivers may inform professional development and obesity-prevention strategies in childcare settings. This cross-sectional validated online questionnaire aimed to measure current early childhood education and care (ECEC) teachers’ nutrition knowledge for pre-schoolers (2–5-year-olds) and related perspectives. Teachers’ (*n* = 386) knowledge of nutrition was lacking: The overall score was 22.56 ± 2.83 (mean ± SD), or 61% correct. Increased years of experience significantly predicted an increase in knowing that national nutrition and physical activity guidelines exist (B = 0.02 [95% CI, 0.00–0.03], *r*^2^ = 0.13, *p* = 0.033). Teachers’ increased agreement in feeling they were confident talking about nutrition to parents significantly predicted an increase in overall nutrition knowledge scores (B = 0.34 [95% CI, 0.06–0.63], *r*^2^ = 0.15, *p* = 0.019). The belief that ECEC teachers play a vital role in promoting pre-schoolers’ healthy eating and physical activity was widespread. Common knowledge barriers included a lack of staff training, confidence, and resources. ECEC teachers may lack nutrition knowledge for pre-schoolers, particularly in regard to basic nutrition recommendations (servings, food/beverage choices, and portion sizes).

## 1. Introduction

With more children being enrolled in early childcare education and care (ECEC) centres [1,2], this setting is becoming increasingly important for children’s health and body weight [3,4,5]. Despite conflicting research [6,7], many studies suggest that children who attend childcare are more likely to be overweight or obese [8,9,10]. Obesity in childhood has immediate and long-term health consequences [11,12] and strongly predicts adult obesity [13,14,15]. 

Global strategies to eliminate childhood obesity recognise the importance of caregivers’ knowledge [16] and their understanding of the links between health, diet, and physical activity in children [17]. Although there is mixed evidence, a systematic review found that nutrition knowledge may have a role in establishing healthier food habits in children [18]. ECEC teachers’ knowledge about nutrition and physical activity for pre-schoolers (2–5-year-olds) remains relatively unreported. Most studies have been conducted amongst ECEC teachers in the US [19,20,21,22,23,24,25,26,27,28,29,30,31,32,33], who likely differ from those living in other countries in regard to ethnic and educational background; for example, important New Zealand ethnic groups (e.g., Māori, Pacific peoples, and Asian) are not distinguished amongst US ECEC teachers [34]. Acknowledging the effects of these variables are important with studies showing that ethnic [26] and educational backgrounds affect nutrition knowledge [28,33]. Moreover, there is considerable variability across studies, in regard to questionnaire content and psychometric validity, and it appears that the measurement of knowledge about important nutrition concepts (e.g., portion sizes and drink choices) is limited. Previous studies using knowledge questionnaires that demonstrate at least construct and content validity are relatively out-of-date (ranging from 1980 to 2015) [22,26,28,29], and the most comprehensive appear to reflect knowledge during the 1970s to the 1990s [29,30,33]. A recent study of 222 caregivers’ knowledge in China, although comprehensive, used a knowledge questionnaire that was lengthy (45 min to complete), focused on nutrients (rather than foods), and was partially validated (only internal reliability assessed) [35]. Three other recent studies used partially validated questionnaires to measure caregivers’ nutrition knowledge, which either lacked comprehensiveness and/or included content that may require highly specific knowledge (e.g., nutrients, oral hygiene, or principles from a specific food program) [19,20,36]. 

In view of these limitations, the available evidence demonstrating a lack of nutrition and physical activity knowledge amongst caregivers remains suggestive [19,21,22,24,28,30,33,35,37,38]. Reports of high levels of nutrition knowledge should be reviewed with caution, as these findings are often based on questionnaires that lack validity or specificity. For example, Halloran et al. [20] identified high nutrition knowledge scores amongst US teachers, but knowledge questions related to nutrition for adults (emphasising specialised topics, rather than basic healthy food-eating principles) and demonstrated content validity only. The relatively small sample size (*n* = 85) weakened generalisability of results. It is important to confirm what knowledge gaps exist, in order to design and justify interventions providing relevant training that will give teachers the confidence and abilities to support children’s healthy eating and physical activity. The aim of this cross-sectional study was to measure what ECEC teachers know about nutrition for pre-schoolers and to investigate their nutrition and physical-activity perspectives. These results could make a valuable contribution to the current literature describing childcare teachers’ early life nutrition knowledge. Such information could be useful for the development of international goals that work toward creating supportive environments for children’s health. 

## 2. Materials and Methods 

### 2.1. Participants

A cross-sectional anonymous online questionnaire was used to collect data from ECEC teachers employed by New Zealand ECEC centres, which is any group-based education and care setting for children under 5 years old (under-5s) (e.g., day cares, nurseries, preschools, and kindergartens) [39,40]. To meet study objectives, only ECEC teachers who held an approved New Zealand ECEC teacher’s qualification (certificate, diploma, bachelors, or higher) and/or had a role as a qualified teacher were selected. To improve specificity, participants who did not hold an ECEC qualification or did not specify the field of their qualification were excluded if they also did not select “qualified teacher” as their current role. Ethical approval to conduct this study was provided by the Massey University Human Ethics Committee: Northern (application 15/36).

### 2.2. Recruitment

Invitations to participate in the study were primarily sent by email to the manager, administrator, or head teacher of New Zealand ECEC centres. This invitation included a link to the questionnaire via the online software tool Qualtrics, which could be accessed from 16 May to 7 August 2017. The link was available on several media platforms (e.g., social media, web applications, and mass media). Approximately two-to-four email reminders were sent. No incentives were offered to participants. Informed consent was questionnaire submission. 

### 2.3. Questionnaire 

**Figure 1:**
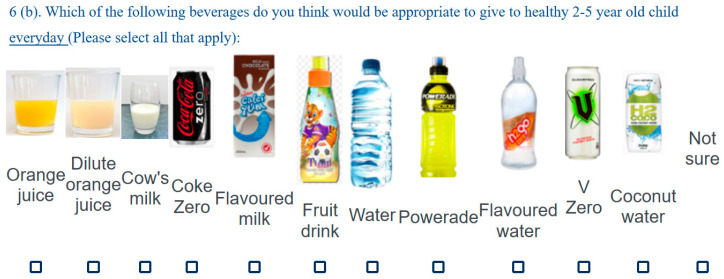
Example item from the food-choices section of the knowledge questionnaire.

The validated 40-item questionnaire took approximately 15 min to complete. Items were based on New Zealand nutrition and physical activity guidelines for pre-schoolers [41,42], literature studies, and an expert panel including three key ECEC stakeholders and three nutrition and exercise science experts. Nutrition-knowledge items were grouped into the subcategories servings, food choices, portions, and resources, which directly measured nutrition knowledge. Serving-size examples [43] were provided to standardise answers for the servings’ subcategory only. Food-portion images [44] and other food/beverage images were included to improve readability (Figure 1). Including a combination of closed- and open-ended questions aimed to maximise data quality and increase response rates [45]. A mixture of forced responses and request responses aimed to minimise missing data; options to “choose not to answer” were included. A correct response was given a +1, whereas an incorrect, “choose not to answer”, or unsure response scored a 0; the sum of these provided the total and subcategory scores. Nineteen items objectively measured nutrition and were organised into the subcategories: servings (6 questions; maximum score 6), food choices (7 questions; maximum score 25), portions (5 questions; maximum score 5), and resources (1 question; maximum score 1). Participants could score a maximum total of 37. The higher the score, the better the performance. 

To ensure acceptable difficulty, relatively basic nutrition principles were tested. Recommended daily intakes (RDI) were excluded, as these are historically difficult concepts to grasp [24,33,46,47] and feature less in public nutrition resources [43]. After feedback from the expert panel, items were omitted or adjusted. The nutrition-knowledge items satisfied psychometric criteria of validity (content and construct) and test-retest reliability [48]. Perspective and demographic items were included to enrich data and were measured by using Likert scales, multi-select boxes, and/or free text.

Responses were scored to attain overall and subcategory mean nutrition-knowledge scores. Although previous studies have interpreted an overall nutrition-knowledge score of ≤61% as low [22,23,29], formal criteria were not explained, and study designs varied; therefore, criteria for high, medium, or low scores could not be deduced from the literature. To be conservative and reduce subjectivity, scores below 100% indicated knowledge deficit; the further below 100%, the greater the knowledge deficit. Subcategory scores were compared to each other, using the terms “higher” or “lower”. To align with previous studies [26,49], perspectives were regarded as positive if they supported current nutrition or physical-activity guidelines, negative if they were unsupportive, or mutual if they indicated “neither agree nor disagree”.

### 2.4. Statistical Analysis

Statistical analysis was conducted by using IBM SPSS Statistics (version 24.0). Descriptive statistics summarised participant characteristics and mean ± SD overall and subcategory nutrition-knowledge scores. As only 1.3% of the sample was male, no gender analysis was undertaken. One-way analysis of variance (ANOVA) was used to assess if nutrition-knowledge scores were affected by teacher variables. Post hoc tests, using Bonferroni correction, determined where these differences lay. Statistical significance was set at *p* < 0.05. Linear regression analysis was conducted to predict nutrition knowledge scores from participant variables. Qualitative information did not require statistical analysis.

## 3. Results

### 3.1. Participants

**Figure 2:**
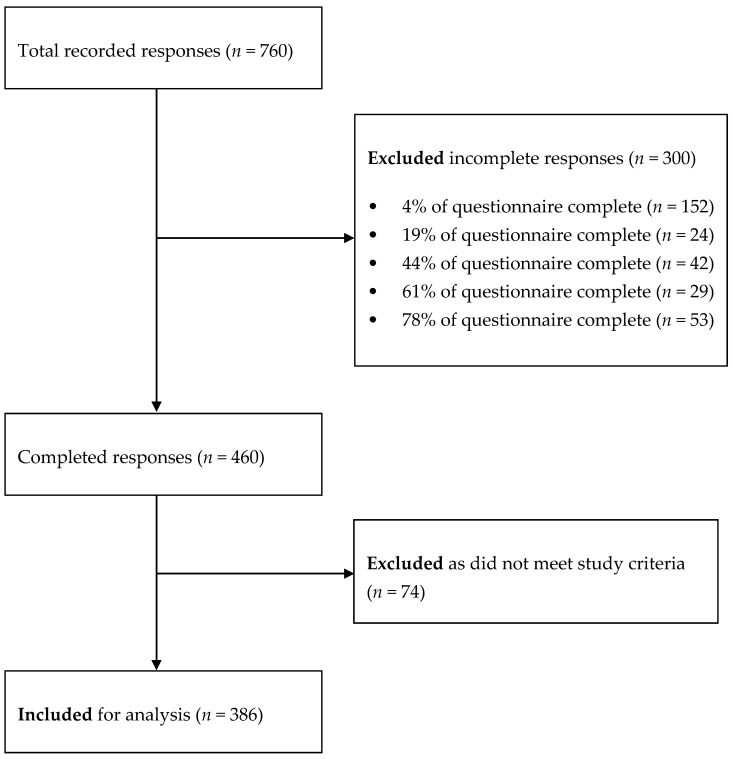
Participant flow diagram. Demographic information for incomplete responses was unavailable.

A total of 386 ECEC teachers were eligible and included in the study (Figure 2). The population comprised 328 female and five male teachers (53 chose not to answer), aged 39.9 ± 11.5 years, with a range of ethnicities and from all regions of New Zealand (Table 1). Of the total responses, 88.0% indicated ECEC-related qualifications and 94.0% stated they were currently in a teaching role; those who did not specify their current role held ECEC-related qualifications. The teachers’ years of experience was 7.3 ± 4.2 years.

### 3.2. Overall Nutrition Knowledge

Teachers’ overall and subcategory mean nutrition-knowledge scores are shown in Table 2; responses for each item are shown in Table 3. The overall mean score was 22.56 ± 2.83, or 61.1% correct; the lowest score was 13.5, and the highest was 30. Differences in participant characteristics (e.g., age, qualification, current role, or years of experience) did not significantly affect overall nutrition-knowledge scores.

### 3.3. Servings

The mean servings’ score was 2.26 ± 1.16, or 43.3% correct (Table 2); 18 teachers scored 0, and one teacher answered all six items correctly. Examples of incorrect answers included “at least 3” (*n* = 172) and “at least 7” (*n* = 1) servings of vegetables, no breads and cereals (*n* = 4), and “at least 7” (*n* = 1) servings of meat/meat alternatives; 49 responses were “not sure”. There was a small but significant effect of teachers’ years of experience on servings’ scores (F (2, 383) = 3.93, *p* < 0.05, d = 0.3). Teachers’ years of experience impacted on servings’ scores (*p* = 0.02), with the 4–10-years group (2.45 ± 1.2) scoring higher than the ≥ 11-years group (2.12 ± 1.1, *p* < 0.05); there was no difference between the ≤3 years’ group and other groups. Correlational analysis showed no relationship between years of experience and servings’ score (*r* = -0.02, *p* = 0.633). No other variables significantly affected serving scores.

### 3.4. Food Choices

The mean food choices score was 18.03 ± 2.24, or 72% correct (Table 2); eight teachers scored less than 50% correct, and no teachers scored all items correctly. When asked to specifically identify suitable everyday drinks from a given pictorial list (Figure 1), items incorrectly selected were dilute orange juice (*n* = 79; 20.5%), coconut water (*n* = 60; 15.5%), Calci-yum flavoured milk (*n* = 15; 3.9%), flavoured water (*n* = 11; 2.8%), orange juice, (*n* = 7; 1.8%), and fruit drink (*n* = 6; 1.6%); none selected Coke Zero, Powerade, and V-Zero. When asked to use free text to describe any other suitable drinks, answers included cow’s milk alternatives (e.g., almond, soya, goats, and oat), breast milk, herbal tea, fruit/vegetable smoothies, kombucha/probiotics, freshly squeezed juice, toddler milk, and milk-flavoured drink (Milo); one teacher commented that “energy drinks” were suitable.

Specific incorrect answers for nuts (any type) included every day (*n* = 170; 44.0%), sometimes (*n* = 145; 37.6%), and occasional (*n* = 32; 8.3%); 15 (3.9%) teachers were unsure and (*n* = 1; 0.3%) chose not to answer. Incorrect answers for hard dried fruits included every day (*n* = 31; 8.0%), sometimes (*n* = 210; 54.4%), and occasional (*n* = 111; 28.8); eight (2.1%) teachers were unsure. Incorrect responses for snack frequency included at least four (*n* = 25; 6.5%), at least five (*n* = 6; 1.6%), at least six (*n* = 8; 2.1%), and more than six snacks (*n* = 2; 0.5%) per day; 13 (3.4%) teachers were unsure, and two (0.5%) chose not to answer. When asked to provide free text to describe other suitable snacks, incorrect answers included mostly small hard foods (e.g., nuts and large seeds) or high salt, saturated fat, or sugar foods (e.g., pretzel, dried fruit, and muesli bars). Items selected for a healthy lunchbox (in different combinations) were cheese (*n* = 363; 94.0%), wholemeal bread (*n* = 369; 95.6%), tomato (*n* = 379; 98.2%), mandarin (*n* = 379; 98.2%), lettuce (*n* = 365; 94.6%), fruit-and-nut muesli bar (*n* = 73; 18.9%), fruit roll-up (*n* = 7; 1.8%), fried chicken nuggets (*n* = 5; 1.3%), and potato crisps (*n* = 5; 1.3%). Teacher variables did not significantly affect food-choice scores. 

### 3.5. Portions

The overall mean portions’ score was 1.77 ± 1.41, or 36% correct (Table 2); 89 teachers did not answer any items correctly, and 15 teachers answered all five items correctly. Teacher variables did not significantly affect portion scores. One teacher commented that “there can be a significant difference between healthy portion sizes for a 2-year-old and a near 5-year-old”.

### 3.6. Resources

The overall mean resources score was 0.51 ± 0.40 correct, or 50% correct (Table 2); 112 teachers scored 0, and 127 teachers answered both items correctly. There was a small but significant effect of teachers’ years of experience on resources scores (F (2, 383) = 5.68, *p* < 0.01, *d* = 0.17). Teachers’ years of experience impacted on servings’ scores (*p* = 0.004), with the ≥11 years group (0.58 ± 0.39) scoring higher than those with ≤3 years (0.41 ± 0.42, *p* < 0.05, d = 0.42) and 4–10 years (0.47 ± 0.40, *p* < 0.05, d = 0.28) of experience; there was no difference between the 4–10-years and ≤3-years groups. Correlational analysis showed a significant relationship between years of experience and resource score, but the effect size is small (*r* = 0.16, *p* = 0.001). Regression analysis showed that increased years of experience significantly predicted an increase in awareness of New Zealand nutrition and physical-activity guidelines (B = 0.02 [95% CI, 0.00–0.03], *r*^2^ = 0.13, *p* = 0.033). Other teacher variables did not significantly affect resources’ scores.

### 3.7. Perspectives

ECEC teachers’ nutrition- and physical-activity-related perspectives can be viewed in Table 4. Increased agreement in teachers feeling confident in talking about nutrition significantly predicted an increase in overall nutrition knowledge scores, (B = 0.34 [95% CI, 0.06–0.63], *r*^2^ = 0.15, *p* = 0.019); no other significant relationships were found between nutrition perspectives and nutrition knowledge. Teachers reported “I am able to define the term physical activity” (98.2%) and “fundamental movement skills” (90.4%), “I understand the difference between structured and unstructured play” (98.4%), “I know what activities will develop specific movements skills” (93.8%), and “I have the skills and abilities I need to support children’s physical activity and development” (96.1%). One teacher commented that “many teachers are unaware of the fundamentals” of physical activity.

### 3.8. Barriers for Teachers’ Knowledge

Teachers stated “no, there are no factors that make it difficult to know about healthy eating” (42.0%) or “physical activity” (52.0%). Commonly perceived barriers for nutrition knowledge were confusing nutrition messages in the media (32.0%), lack of staff training (19.0%), lack of confidence talking about nutrition (14.0%), lack of support from parents/whānau (13.2%), and lack of resources (12.0%); one teacher specified a barrier of “inappropriate advice promoting diets high in carbohydrate and sugar, e.g., the food triangle”. Common barriers for teachers’ physical-activity knowledge included lack of resources (21.0%), lack of staff training (16.1%), lack of confidence (12.7%), and insufficient time to teach physical activity (11.4%); four teachers indicated safety concerns. 

### 3.9. Additional Comments

Collaboration with parents/whānau (family) to support pre-schoolers’ healthy eating was a key theme amongst teachers’ comments. Comments included “It’s a very ‘hairy’ issue for ECEC teachers (who are we to judge the parents?)” and “it is really about getting parents and whānau on board”. Five teachers expressed a desire for more-accessible, less-expensive, more-interactive (e.g., games) or easier-to-understand nutrition/physical-activity resources. 

## 4. Discussion

This study investigated ECEC teachers’ nutrition and physical activity knowledge for pre-schoolers (2–5-year-olds). The findings indicate a lack of nutrition knowledge amongst New Zealand ECEC teachers. Teachers generally reported that they understood key physical-activity concepts; however, they desired more physical-activity training. 

### 4.1. Overall Nutrition-Knowledge Scores

The overall nutrition knowledge score of 61% suggests a lack of nutrition knowledge amongst New Zealand ECEC teachers. This is in agreement with previous studies in other countries reporting similar deficits, with overall nutrition-knowledge scores ranging from 51% to 67.1% correct [19,21,22,23,29,33,35]. Similar to Jones et al. [22], we view this score as less than ideal, especially since teachers lacked knowledge about relatively basic nutrition concepts (recommended servings, food choices, and portion sizes). 

### 4.2. Servings

A mean servings’ score of 43.3% correct answers suggests that ECEC teachers lack knowledge about the recommended servings from the four main food groups, for pre-schoolers. Knowledge deficits were apparent across all food groups; however, teachers scored the lowest for breads and cereals, so they may lack the most knowledge about this food group. Most (84.9%) teachers underestimated the recommended servings for breads and cereals, while 42% incorrectly answered that pre-schoolers must eat wholegrains every day. Likewise, Rida et al. [19] found all participants scored low on wholegrain items that should be served daily; only 43.3% and 45.6% of urban and rural participants, respectively, correctly identified the amount of wholegrain-rich items that should be served every day. Pre-schoolers that consume too-little breads and cereals might not meet their energy and B-vitamin requirements for rapid brain development and growth, or dietary fibre for digestive health [41]. Meanwhile, pre-schoolers consuming too many wholegrain foods may experience early satiety (due to their small stomachs), and thus find it difficult to meet their high energy requirements. These risks highlight the importance of ensuring that teachers understand dietary guidelines for breads and cereals. Perhaps knowledge deficits in regard to food-group servings are due to confusing media messages (e.g., low-carbohydrate diets), with 32% of teachers identifying this to be a barrier for their nutrition knowledge. Similarly, Sharma et al. [24] reported that over half of ECEC teachers found it difficult to know what nutrition information to believe; few (10.0%) teachers were able to correctly answer for grains, while more teachers (68.0%) correctly answered for fruits and vegetables. Teachers also scored higher for fruits and vegetables than breads and cereals in our study, yet 45.6% and 70.6% of teachers, respectively, indicated a greater number of servings than is recommended for fruits and vegetables. Perhaps teachers are overestimating pre-schoolers’ serving requirements after misinterpreting prominent health-promotion messages, such as 5+ a Day [50]. Teachers may know more about recommended servings for lean meat/meat alternatives and milk/milk alternatives. However, with 49% and 33% of teachers providing incorrect answers for each, respectively, there is room to improve their knowledge. Mixed results for the relationship between years of experience and servings’ score may be an effect of high inter-individual variation (SD = 1.16) between servings’ scores, so future studies may clarify findings. Nevertheless, if ECEC teachers lack knowledge about recommended daily servings, it seems difficult to expect them to support pre-schoolers in meeting their daily energy and nutrient requirements.

### 4.3. Food Choices

Less than 10% of teachers knew that whole nuts and hard dried fruits should never be given to under-5s. These small hard foods are choking hazards [41]; thus, a lack of knowledge may be a health and safety issue that should be immediately addressed. Meanwhile, although over 60% were able to correctly identify occasional foods (e.g., pastries and ice-cream), up to 17% of teachers stated that these should never be eaten. Demonisation of high fat/sugar foods in the media [51,52] and ECEC nutrition policies that misalign with Ministry of Health guidelines [53] may be contributing to these knowledge deficits. Since banning foods can encourage children to fixate on these items and consume them in excess [54,55], it is important that teachers have the knowledge and skills to follow guidelines for food choices. Meanwhile, although 80% of teachers knew the recommended number of snacks per day, 10.7% of teachers answered more than is recommended. Too much snacking may encourage grazing habits, which is associated with dental caries [41,56] and problematic or disordered eating behaviours [57]. Teachers may be unaware of this or are unclear on what constitutes too much snacking. Teachers may have better knowledge about suitable drinks for pre-schoolers, since ≥80% of teachers answered almost all drink items correctly. However, only 55% identified that cow’s milk and water were the only recommended everyday drinks; and three teachers did not know that sports/energy drinks should never be consumed. Similarly, Rida et al. [19] found only 51.4% and 58.9% of urban and rural caregivers correctly identified suitable milk drinks for children in their childcare centre, respectively. This is the first study, to the authors’ knowledge, to objectively measure and report ECEC teachers’ knowledge on a range of available drinks and snacks for pre-schoolers. Findings suggest that New Zealand ECEC teachers lack knowledge about recommended food choices for pre-schoolers. If teachers are unable to decipher between “everyday”, “occasional”, and “never” foods or drinks, they may teach, provide, and/or role model inappropriate food choices to pre-schoolers, and thus unwittingly encourage pre-schoolers to establish harmful dietary patterns.

### 4.4. Portions 

With a mean portions’ score of only 36% correct, ECEC teachers may lack understanding about the difference between a portion and a serving (even after viewing examples of a serving in an earlier section of the questionnaire). It is important that teachers understand that a serving size is not the same as a portion size. According to Ministry of Health guidelines [41], a portion is defined as the amount of food offered at a single eating occasion, whereas a serving is a standard measured amount and does not vary according to the size of an individual’s hand. Although knowledge deficits were widespread in this subcategory, teachers seemed to have the most difficulty identifying a portion of meat (24% correct). Some teachers believed that food amounts or portion sizes differ greatly between a two-year-old and a five-year-old; however, a recent UK study on evidence-based portion sizes showed that a 100–120 mL portion of milk is suitable and adequate for healthy 1–4-year-olds [44]. Similarly, difficulties in identifying the correct serving size for milk have been reported amongst US teachers [19]. Our study is one of few to examine ECEC teachers’ knowledge about portions/serving sizes; however, a common misconception is that a serving size for a child is the amount which roughly fits the size of a child’s hand [58,59,60]. If teachers misunderstand these concepts, they may not be able to support children in meeting their recommended servings per day. As portion sizes continue to grow across food sources (potentially contributing to childhood obesity) [61,62,63], teachers may need more support to determine healthy portion sizes for pre-schoolers.

### 4.5. Resources

Only 56% of teachers knew about the Ministry of Health food and nutrition guidelines for pre-schoolers, and fewer (46%) teachers knew that there were New Zealand physical-activity guidelines for pre-schoolers. This may be expected, as more comprehensive physical-activity guidelines [64] were only released at the time of data collection, whereas Ministry of Health nutrition guidelines have been available since 2012. Nevertheless, if teachers do not know that these resources exist, they cannot be expected to know about the recommendations. Additionally, >70% of those who knew of these guidelines also said they used them, which seems at odds with the apparent knowledge deficits. This, along with at least 14% not using the guidelines, suggests that teachers need further support in knowing about and then using these resources. 

### 4.6. Determinants of ECEC Teachers’ Nutrition Knowledge

Overall, nutrition-knowledge scores were not significantly affected by age, qualification level, employment role, or years of experience. This is in agreement with previous studies reporting no relationship between US ECEC teachers’ educational background and/or previous nutrition training [19,29,33]; however, it disagrees with other studies showing that ECEC teachers with higher qualifications and/or who were teaching nutrition had significantly better nutrition knowledge scores [28,33]. Liu et al. [35] found several teacher characteristics predicted knowledge scores, including age, type or residence, body mass index, and education background. These equivocal findings may be due to considerable variability between study methodologies (e.g., questionnaires design), or our findings may be unique to New Zealand ECEC teachers. Nevertheless, results suggest that nutrition-knowledge deficits may be widespread amongst ECEC teachers in New Zealand, regardless of their background. 

Subcategory scores were mostly unaffected by differences in teacher variables. It seems that teachers with more years of experience may be more aware that there are New Zealand nutrition and physical-activity guidelines. Reasons for this seem unclear; however, a US study found that ECEC teachers with more years of experience (6–10 years) reported nutrition/physical-activity government guidelines as being less helpful [27]. It was suggested that less-experienced teachers needed to rely on guidelines more often, so they found them more helpful. Perhaps our findings indicate that less-experienced teachers have had less opportunity to be exposed to nutrition and physical-activity guidelines, resulting in lower scores. Overall, this evidence suggests that less-experienced teachers may need more support in knowing about current nutrition and physical-activity guidelines, and perhaps teachers with more experience could offer some of this support. 

### 4.7. Perspectives and Barriers

The belief that ECEC teachers play a vital role in supporting children’s healthy eating and physical activity was widespread amongst teachers, which is consistent with previous research [24,27,28,37,38,65]. Teachers generally held positive feeding practice perspectives; however, responses were mixed as to whether “pre-schoolers should always eat everything on their plate”. This statement references guidelines to avoid forcing, pressuring, or bribing children to eat [41] and is commonly argued amongst ECEC teachers [25,26]. It is important that teachers are aware that coercive feeding practices are not recommended, as they are associated with poor dietary behaviours and increased body weight in pre-schoolers [66,67,68,69,70]. As to whether these mixed views indicate knowledge deficits or simple disagreement amongst ECEC teachers must be confirmed by objective measures. Nevertheless, widespread supportive nutrition perspectives amongst ECEC teachers seem promising for creating supportive ECEC nutrition environments. 

ECEC teachers generally believed that they were able to define and understand key physical-activity concepts, and they thought that they had the knowledge, skills, and abilities to support these. However, conflicting comments, such as “many teachers are unaware of the fundamentals [of physical activity]”, may suggest that teachers still lack physical-activity knowledge. This may be supported by findings from a recent New Zealand intervention study that identified a need to increase ECEC teachers’ physical-activity knowledge, with few teachers being able to identify relevant theory or research related to physical activity (pre- and post-intervention) [71]. Furthermore, knowledge gaps seem possible, with teachers desiring more staff training, resources, and confidence in physical activity in childcare and identifying a lack of these to be barriers to their physical-activity knowledge. This study is the first to specifically report barriers for ECEC teachers’ nutrition and physical-activity knowledge; however, a desire and need for more training has previously been reported [22,35,65,71,72]. For example, McLachlan et al. [71] found that some (*n* = 8) ECEC teachers identified that they needed to learn more about how to intentionally teach physical activity and wanted more physical-activity resources. It was also suggested that further support was needed to increase teacher knowledge and confidence about nutrition and physical activity in childcare [71]. Overall, these findings may further justify providing teachers with physical-activity professional development. However, objective measures of physical activity knowledge are needed to accurately guide training content. 

Most teachers felt confident talking to parents/whānau about nutrition, which seems to be an improvement on the lack of confidence previously reported by a *Healthy Heart Award* evaluation [72]. However, teachers in our study still perceived a lack of staff confidence in regard to talking about nutrition or teaching physical activity as a barrier for their nutrition or physical-activity knowledge. Teachers were also concerned about how to effectively collaborate with and get parents “on board” with supporting children’s healthy eating and physical activity. Perhaps addressing knowledge deficits toward basic nutrition concepts (as identified in this study) and providing relevant training may give teachers the confidence they need to work with families in supporting pre-schoolers’ nutrition and physical activity. Improving ECEC teachers’ confidence to talk with families may be especially important if this predicts increases in nutrition knowledge.

Overall, this evidence may suggest that teachers are missing key elements of “nutrition literacy” [73] and “physical activity literacy” [74], which are concepts that link knowledge, confidence, and skills with dietary or physical activity decision/actions, and are strong predictors of health [73,74,75]. Interventions aiming to establish adequate nutrition and physical-activity literacy amongst New Zealand ECEC may find targeting the specific knowledge gaps identified in this study useful. 

### 4.8. Limitations and Strengths

The main limitation of this research is the potential for subjective analysis (e.g., researchers or subjects interpreting items differently), yet this bias was likely reduced by using a psychometrically valid questionnaire and a standard measure to score items. Social desirability may have skewed results for perspectives. The online questionnaire format may have excluded less well-resourced centres, and participants may have volunteered based on their interest in the research topic, thus skewing results and limiting generalisability. However, the relatively large sample size and diversity of teachers’ ethnic and geographical location may have reduced bias. Finding out if participants had previous nutrition training may have enriched discussion and identified causes for knowledge deficits; however, identifying causes and effects was beyond the scope of this study. A key strength of this study is that it investigated ECEC teachers’ nutrition and physical-activity knowledge for pre-schoolers by using a psychometrically valid knowledge questionnaire, thus helping to address the limitations of previous research. 

## 5. Conclusions

ECEC teachers may lack nutrition knowledge for pre-schoolers, particularly in regard to relatively basic nutrition recommendations (servings, food/beverage choices, and portion sizes). While these findings are specific to a New Zealand context, they align with those of other countries, thus building on the current evidence base for ECEC teachers’ nutrition knowledge. In addition, this study may be of interest to those wishing to conduct further assessments of caregivers’ knowledge. Future interventions may provide training, resources, or policy changes that target these knowledge gaps and help teachers improve skills and confidence, to support children’s healthy eating and physical activity. This should be received well if teachers believe they have a role in promoting nutrition and physical activity to pre-schoolers and desire more nutrition and physical-activity resources and training.

## Figures and Tables

**Table 1:** Participant characteristics (*n* = 386).

	*n*	%
Gender (*n* = 333) ^1^		
Male	5	1.3
Female	328	85.0
Age, years, (*n* = 377) ^1^		
20–29	94	24.4
30–39	102	26.4
40–49	96	24.9
50–59	61	15.8
60–69	24	6.2
Ethnicity ^2^		
Māori	39	9.1
Pacific Island	15	3.9
New Zealand European/Pakeha	280	72.5
Other European	52	13.5
Asian/Indian	61	15.8
Qualification level		
Sub-degree (certificate, diploma)	128	33.2
Bachelor	217	56.2
Post-graduate level	31	8.0
Other	10	2.6
Employment role		
Qualified teacher	319	82.6
Manager/teacher	44	11.4
Other (+teachers’ qualification)	23	3.1
Years of experience		
≤3	60	15.5
4–10	163	42.2
≥11	163	42.2
ECEC region ^1^		
Auckland	90	23.3
Bay of Plenty	25	6.5
Canterbury	62	16.1
Hawkes Bay	3	0.8
Manawatu-Wanganui	11	2.8
Marlborough	3	0.8
Nelson	1	0.3
Northland	4	1.0
Otago	22	5.7
Southland	8	2.1
Taranaki	11	2.8
Tasman	1	0.3
Waikato	44	11.4
Wellington	45	11.7

^1^ The remaining participants chose not to answer. ^2^ Does not total 100% because participants could select more than one option. Note: ECEC, early childhood education and care.

**Table 2:** Nutrition knowledge scores by participant variables.

	Total Score (Mean ± SD)	Servings Score (Mean ± SD)	Food-Choices Score (Mean ± SD)	Portions Score (Mean ± SD)	Resource Score (Mean ± SD)
All teachers (*n* = 386)	22.56 ± 2.83	2.26 ± 1.16	18.03 ± 2.24	1.77 ± 1.41	0.51 ± 0.40
Variable					
Age, years					
20–29 (*n* = 94)	22.16 ± 2.82	2.28 ± 1.09	17.73 ± 2.44	1.78 ± 1.38	0.45 ± 0.40
30–39 (*n* = 102)	22.71 ± 2.60	2.32 ± 1.18	18.03 ± 2.14	1.80 ± 1.41	0.49 ± 0.40
40–49 (*n* = 96)	22.45 ± 3.01	2.40 ± 1.20	18.14 ± 2.29	1.65 ± 1.41	0.50 ± 0.40
50–59 (*n* = 61)	22.84 ± 3.10	1.92 ± 1.08	18.07 ± 2.27	1.93 ± 1.48	0.59 ± 0.40
60–69 (*n* = 24)	23.33 ± 2.42	2.17 ± 1.17	18.63 ± 1.88	1.83 ± 1.34	0.63 ± 0.40
Choose not to answer (*n* = 9)	22.50 ± 2.33	2.33 ± 1.32	18.11 ± 1.05	1.44 ± 1.59	0.50 ± 0.43
Qualification level					
Sub-degree (*n* = 128)	22.27 ± 2.84	2.19 ± 1.18	17.97 ± 2.19	1.73 ± 1.48	0.53 ± 0.41
Bachelors (*n* = 217)	22.75 ± 2.75	2.32 ± 1.12	18.07 ± 2.21	1.82 ± 1.38	0.49 ± 0.40
Post-graduate level (*n* = 31)	22.76 ± 2.78	2.23 ± 1.33	18.39 ± 2.17	1.55 ± 1.23	0.53 ± 0.39
Other (*n* = 10)	21.60 ± 4.14	1.90 ± 0.20	16.70 ± 3.30	1.90 ± 1.60	0.50 ± 0.47
Employment role					
Qualified teacher (*n* = 319)	22.52 ± 2.80	2.29 ± 1.19	18.08 ± 2.22	1.73 ± 1.37	0.51 ± 0.40
Manager/teacher (*n* = 44)	23.00 ± 3.23	2.07 ± 0.97	17.84 ± 2.53	2.05 ± 1.60	0.55 ± 0.42
Other (*n* = 33)	22.30 ± 2.36	2.17 ± 0.94	17.74 ± 2.00	1.78 ± 1.54	0.43 ± 0.43
Years of experience					
≤3 (*n* = 60)	21.91 ± 3.21	2.12 ± 1.22	17.55 ± 2.61	1.62 ± 1.45	0.41 ± 0.42
4–10 (*n* = 163)	22.72 ± 2.75	2.45 ± 1.14 ^1^	18.14 ± 2.15	1.79 ± 1.40	0.47 ± 0.40
≥11 (*n* = 163)	22.65 ± 2.73	2.12 ± 1.12	18.09 ± 2.17	1.82 ± 1.41	0.58 ± 0.39 ^2^

^1^ Statistically significantly greater mean score than teachers with 11+ years of experience (*p* < 0.05). ^2^ Statistically significantly greater mean score than teachers with ≤3 years or 4–10 years of experience (*p* < 0.05). Note: SD, standard deviation.

**Table 3:** Responses of ECEC teachers in New Zealand on nutrition-knowledge items (*n* = 386).

Question Content ^1^	Correct Response	% Correct ^2^
Servings		
Fruit	At least 2	46
Vegetables	At least 2	23
Breads and cereals	At least 4	8
Wholegrain breads/cereals	Most days	31
Lean meat/alternatives	At least 1	51
Milk products/alternatives	At least 2 or 3	67
Food choices: frequency of beverages/snacks		
Water	Everyday	99
Cow’s milk	Everyday	82
Flavoured milk	Occasional/never	90
Fizzy drinks	Occasional/never	99
Cordial or fruit drinks	Occasional/never	95
Tea or coffee	Never	98
Sports or energy drinks	Never	99
Suitable everyday drinks ^3^	Cow’s milk + water	55
Other suitable drinks ^4^	Recommended ^5^	88
Potato/corn crisps	Occasional	63
Fruit	Everyday	99
Chocolate/cream biscuits	Occasional	63
Plain biscuits Yoghurt pottle (all types)	Sometimes/occasional Everyday	93 38
Vegetable sticks	Everyday	95
Nuts	Never	6
Hard dried fruits	Never	8
Soft dried fruits	Sometimes	59
Ice-cream	Occasional	70
Cheese	Everyday	57
Pastries	Occasional	64
Plain crackers/crisp breads	Sometimes	52
Snacks per day	2 or 3 snacks	80
Other suitable snacks	Recommended	80
Foods for a healthy lunchbox	Cheese + wholemeal bread + tomato + lettuce + mandarin	71
Portions		
Dairy	1 cup cow’s milk (250 mL)	65
Fruit	1 medium banana	56
Vegetables	1 medium carrot	63
Breads and cereals	½ slice of bread	50
Meat	1 chicken drumstick	24
Resources		
MoH nutrition guidelines exist	Yes	56
NZ PA guidelines exist	Yes	46

^1^ Correct answers were based on Ministry of Health Food and Nutrition Guidelines for Healthy Children and Young People (2–18 years) for pre-schoolers. ^2^ The percentage of total sample (*n* = 386) that correctly answered the item. ^3^ Participants selected text and images. ^4^ Responses were free-text format. ^5^ As cow’s milk and water are recommended as the most suitable drinks, an answer of “no” was also correct; listing cow’s milk, water, or alternative milk was marked correct. Notes: MoH, Ministry of Health; NZ, New Zealand; PA, physical activity.

**Table 4:** ECEC teachers’ nutrition and physical activity-related perspectives (seven-point Likert scale).

	n (%)						
Feeding Practices ^1^	Strongly Disagree	Disagree	Somewhat Disagree	Neither Agree Nor Disagree	Somewhat Agree	Agree	Strongly Agree
Mealtimes should be fun (*n* = 385)	1 (0.3)	3 (0.8)	6 (1.6)	20 (5.2)	64 (16.6)	109 (28.2)	182 (47.2)
Pre-schoolers should eat together (*n* = 386)	2 (0.5)	4 (1)	4 (1)	23 (6)	62 (16.1)	131 (33.9)	160 (41.5)
Snacks should be low in sugar (*n* = 386)	3 (0.8)	0 (0)	1 (0.3)	2 (0.5)	25 (6.5)	107 (27.7)	248 (64.2)
Pre-schoolers should have a choice of a variety of foods at mealtimes (*n* = 385)	1 (0.3)	7 (1.8)	11 (2.8)	19 (4.9)	54 (14)	135 (35)	158 (40.9)
Pre-schoolers should always eat all the food on their plate (*n* = 385)	54 (14)	121 (31.3)	80 (20.7)	49 (12.7)	56 (14.5)	17 (4.4)	8 (2.1)
ECEC teachers should role model healthy eating to pre-schoolers (*n* = 383)	0 (0)	0 (0)	1 (0.3)	3 (0.8)	13 (3.4)	110 (28.5)	256 (66.3)
ECEC teachers should eat with pre-schoolers (*n* = 384)	4 (1)	15 (3.9)	9 (2.3)	47 (12.2)	81 (21)	126 (32.6)	102 (26.4)
ECEC teachers should talk to pre-schoolers about what they are eating (*n* = 385)	1 (0.3)	3 (0.8)	2 (0.5)	7 (1.8)	19 (4.9)	122 (31.6)	231 (59.8)
ECEC teachers should encourage pre-schoolers to try new foods (*n* = 385)	1 (0.3)	0 (0)	1 (0.3)	5 (1.3)	23 (6)	142 (36.8)	213 (55.2)
It is important that pre-schoolers are involved in an edible garden in ECEC settings (*n* = 385)	1 (0.3)	0 (0)	0 (0)	13 (3.4)	52 (13.5)	143 (37)	176 (45.6)
**Nutrition-related perspectives ^1^**							
I feel confident having conversations with parents about food and nutrition (*n* = 377)	2 (0.5)	5 (1.3)	17 (4.4)	21 (5.4)	83 (21.5)	132 (34.2)	117 (30.3)
ECEC teachers play a vital role in promoting nutrition to pre-schoolers (*n* = 385)	0 (0)	1 (0.3)	1 (0.3)	7 (1.8)	25 (6.5)	140 (36.3)	211 (54.7)
A pre-schooler’s healthy eating is more of the parent’s responsibility (*n* = 386)	14 (3.6)	36 (9.3)	46 (11.9)	75 (19.4)	108 (28)	66 (17.1)	41 (10.6)
**Physical activity perspectives ^1^**							
ECEC teachers play a vital role in promoting pre-schoolers’ physical activity and development (*n* = 385)	1 (0.3)	0 (0)	1 (0.3)	3 (0.8)	17 (4.4)	113 (29.3)	250 (64.8)
A pre-schooler’s physical activity and development is more of the parent’s responsibility (*n* = 384)	27 (7.0)	63.0 (16.3)	54 (14.0)	112 (29.0)	64 (16.6)	36 (9.3)	28 (7.3)

^1^ Remaining responses were “not sure” or “choose not to answer”.
